# KIT Is Required for Fetal Liver Hematopoiesis

**DOI:** 10.3389/fcell.2021.648630

**Published:** 2021-07-29

**Authors:** Alessandro Fantin, Carlotta Tacconi, Emanuela Villa, Elena Ceccacci, Laura Denti, Christiana Ruhrberg

**Affiliations:** ^1^UCL Institute of Ophthalmology, University College London, London, United Kingdom; ^2^Department of Biosciences, University of Milan, Milan, Italy; ^3^Department of Experimental Oncology, IEO, European Institute of Oncology IRCCS, Milan, Italy

**Keywords:** KIT, hemogenic endothelium, erythromyeloid progenitors, yolk sac, fetal liver

## Abstract

In the mouse embryo, endothelial cell (EC) progenitors almost concomitantly give rise to the first blood vessels in the yolk sac and the large vessels of the embryo proper. Although the first blood cells form in the yolk sac before blood vessels have assembled, consecutive waves of hematopoietic progenitors subsequently bud from hemogenic endothelium located within the wall of yolk sac and large intraembryonic vessels in a process termed endothelial-to-hematopoietic transition (endoHT). The receptor tyrosine kinase KIT is required for late embryonic erythropoiesis, but KIT is also expressed in hematopoietic progenitors that arise via endoHT from yolk sac hemogenic endothelium to generate early, transient hematopoietic waves. However, it remains unclear whether KIT has essential roles in early hematopoiesis. Here, we have combined single-cell expression studies with the analysis of knockout mice to show that KIT is dispensable for yolk sac endoHT but required for transient definitive hematopoiesis in the fetal liver.

## Introduction

Several consecutive waves of hematopoietic progenitors arise and contribute blood and immune cells to the growing vertebrate embryo in close spatiotemporal proximity to developing blood vessels (Hoeffel and Ginhoux, 2018). The first primitive hematopoietic precursors originate in the yolk sac blood islands between E7.0 and E8.25 in the mouse and differentiate into embryonic erythrocytes and yolk sac macrophages (Ginhoux and Guilliams, 2016; Hoeffel and Ginhoux, 2018; Canu and Ruhrberg, 2021). These yolk sac macrophages colonize the embryo and differentiate into tissue-resident macrophages. The transient definitive wave of hematopoietic precursors arises when a subset of ECs in the yolk sac specializes into hemogenic endothelium to undergo an endothelial-to-hematopoietic transition (endoHT) between E8.5 and E9.5 in the mouse (Hoeffel and Ginhoux, 2018). This process generates erythromyeloid progenitors (EMPs), which leave the yolk sac after the onset of blood flow and colonize the liver (Lux et al., 2008; Frame et al., 2013; McGrath et al., 2015; Hoeffel et al., 2015; Hoeffel and Ginhoux, 2018). In the liver, EMPs give rise to both transient definitive erythrocytes and monocyte precursors for tissue macrophages. It is thought that the liver EMP-derived macrophages replace the initial pool of yolk sac-derived macrophages in all organs, with the exception of the tissue-resident macrophages of the central nervous system, termed microglia (Hashimoto et al., 2013; Hoeffel et al., 2015). Finally, the definitive wave of hematopoietic precursors emerges via endoHT in the aorta-gonad-mesonephros (AGM) region (Sanchez et al., 1996; Yokomizo and Dzierzak, 2010) as a continuum of pro-, pre-, and definitive hematopoietic stem cells (HSCs) that seed the liver from E10.5 in the mouse before colonizing the bone marrow just before birth (Taoudi et al., 2008; Rybtsov et al., 2014, 2011; Azzoni et al., 2018).

Hemogenic ECs are induced by retinoic acid signaling, which upregulates the expression of the receptor tyrosine kinase KIT (Dejana et al., 2017), whereby KIT cell surface expression is often used as a distinguishing feature from non-blood-forming ECs (Goldie et al., 2008). Moreover, KIT is also used as a key marker for the progeny of hemogenic endothelium, including EMPs (Gomez Perdiguero et al., 2015; Hoeffel et al., 2015; McGrath et al., 2015) and HSCs (Sanchez et al., 1996; Gomez Perdiguero et al., 2015). Genetic defects that disrupt KIT signaling reduce the number of late embryonic HSCs (Ikuta and Weissman, 1992), affect lymphopoiesis in the adult (Waskow et al., 2002), and cause severe macrocytic anemia and thus late embryonic or perinatal lethality (Bernex et al., 1996; Broudy, 1997; Ding et al., 2012). The anemic phenotype was ascribed to an erythroid differentiation block in the fetal liver after E13.5 (Chui and Russell, 1974; Chui and Loyer, 1975; Chui et al., 1978; Broudy, 1997) that could be rescued by wild-type HSCs (Fleischman and Mintz, 1979) or by erythropoietin overexpression (Waskow et al., 2004). Subsequent studies with function-blocking antibodies further suggested that hematopoietic waves originating before E12.5 depend less on KIT than later embryonic waves (Ogawa et al., 1993). However, it has not been directly addressed whether KIT is required for yolk sac endoHT, EMP formation, or EMP function.

Here, we have combined confocal imaging, single-cell transcriptomic analyses, and flow cytometry to identify the cellular profile of *Kit* expression during early hematopoiesis in the mouse. Our functional studies further show that the hematopoietic requirement for KIT does not comprise yolk sac hematopoiesis, including the generation of EMPs. Instead, we find that KIT is required for EMP expansion and the EMP-dependent process of transient-definitive hematopoiesis that takes place in the fetal liver and includes the generation of fetal erythroid cells. KIT is therefore required for erythropoiesis earlier than previously reported. These conclusions agree with those in prior studies of mice lacking the KIT ligand KITL, also known as stem cell factor (SCF) (Azzoni et al., 2018).

## Results

### KIT Marks EMPs but Is Dispensable for endoHT and Hematopoiesis in the Yolk Sac

**FIGURE 1:**
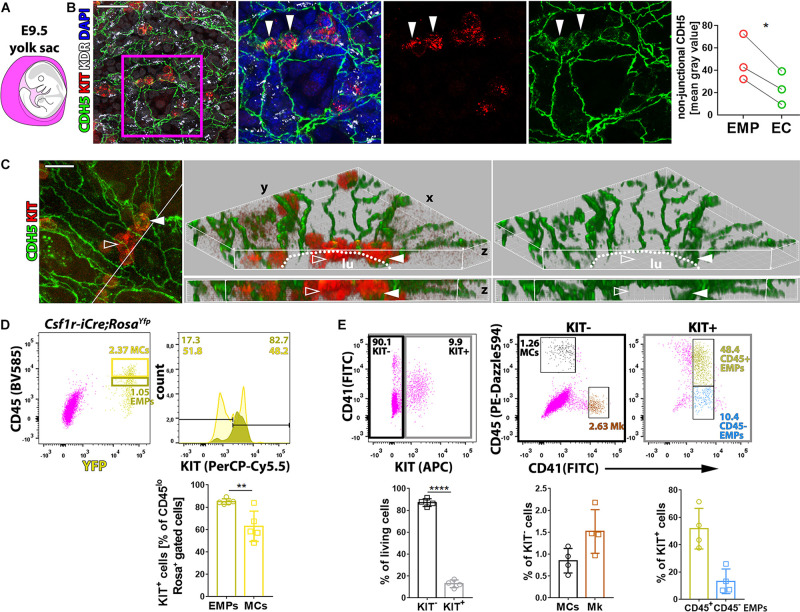
KIT marks EMPs budding from hemogenic endothelium. **(A)** Schematic representation of an E9.5 mouse embryo surrounded by the yolk sac, shown in purple. **(B,C)** Whole-mount labeling of E9.5 mouse yolk sacs with the indicated markers (*n* = 3). The dot plot represents the staining intensity (mean gray value) for CDH5 in the cytoplasm of KIT^+^ EMPs and KIT^–^ ECs; each data point represents the average value from at least seven cells in >2 fields of view from one yolk sac; *p < 0.05 (paired Student’s *t*-test). The white line in the left panel in **(C)** marks the clipping plane for the 3D-rendered lateral views in the center and right panels; white arrowheads indicate KIT^+^ CDH5^+^ budding EMPs, empty arrowheads indicate KIT^+^ CDH5^–^ EMPs in the vessel lumen; the dotted lines indicate the lumen (lu); scale bars: 50 μm **(B)**, 20 μm **(C)**. **(D)** Flow cytometry analysis of single living cells from E9.5 *Csf1r-iCre;Rosa^*Yfp*^or Rosa^*tdTom*^* mouse yolk sacs with the indicated markers (*n* = 5). The dark and bright yellow boxes in the scatter plot indicate the gates used to quantify the percentage of KIT^+^ cells in the populations containing putative EMPs (CD45^low^ YFP^+^ or TdTomato^+^) and myeloid cells (MCs, CD45^high^ YFP^+^ or TdTomato^+^; a presentative experiment is shown for *Rosa*^*Yfp*^), respectively. Pooled data for *Rosa*^*Yfp*^ or *Rosa*^*tdTom*^ experiments are shown as mean ± SD; each data point represents the value from one embryo; **p < 0.01 (paired Student’s *t*-test). **(E)** Flow cytometry analysis of single living cells from E9.5 mouse yolk sacs with the indicated markers (*n* = 4). The black and gray boxes in the scatter plot on the left-hand side indicate the gates used to quantify the proportion of KIT^+^ and KIT^–^ cells, which were further analyzed, as shown in scatter plots in the center and on the right-hand side, respectively. The color-coded annotation indicates the gates used to determine the percentage of the KIT^–^ MCs and megakaryoblasts (Mk) as well as the KIT^+^ CD45^+^ and CD45^–^ putative EMPs.

Whole-mount immunostaining of E9.5 yolk sacs localized KIT to small clusters of cells within the CDH5^+^ KDR^+^ endothelium that appeared rounder and smaller than neighboring ECs, consistent with imminent budding into the vascular lumen (Figures 1A,B). These observations support the idea that KIT is expressed by EMPs generated by hemogenic ECs undergoing endoHT and corroborate that KIT immunostaining distinguishes hemogenic from non-blood-forming ECs. We further found that CDH5 was concentrated at adherens junctions at cell–cell contacts between KIT^–^ ECs, consistent with its key role in joining ECs into vascular channels (Giannotta et al., 2013). Notably, KIT^+^ EMPs budding from the hemogenic endothelium had more intracellular CDH5 staining than neighboring ECs (Figures 1B,C, full arrowhead), and EMPs already released into the vessel lumen appeared negative for CDH5 by immunostaining (Figure 1C, empty arrowhead). These findings suggest that CDH5 internalization precedes EMP budding, presumably as a prerequisite for EMPs to break contact with the endothelial monolayer.

To identify EMPs with flow cytometry, KIT surface expression together with low levels of CD45 staining (CD45^low^) has previously been combined with *Csf1r-iCre*-mediated lineage tracing (Gomez Perdiguero et al., 2015; Hoeffel et al., 2015) or with CD41 co-expression (McGrath et al., 2015; Frame et al., 2016). Using *Csf1r-iCre*-mediated lineage tracing with the recombination reporter *Rosa*^*Yfp*^ or *Rosa^*tdTom*^*, we found that more than 80% of CD45^low^ YFP^+^ or TdTomato^+^ cells were also KIT^+^ in E9.5 yolk sacs (Figure 1D). Analysis of KIT surface expression with both CD41 and CD45 identified a KIT^–^ cell population composed of CD45^+^ CD41^–^ cells (Figure 1E) that likely correspond to myeloid cells (MCs) (McGrath et al., 2015; Frame et al., 2016) and CD45^–^ CD41^+^ cells (Figure 1E) that likely correspond to megakaryoblasts (McGrath et al., 2015; Frame et al., 2016; Cortegano et al., 2019). By contrast, the KIT^+^ population (Figure 1E) included CD41^low^ cells, which were mostly CD45^+^. This cell population likely contains progenitors with definitive erythroid, myeloid, and lymphoid potential, including the previously described *Csf1r*-lineage traced CD45^low^ KIT^+^ EMPs (Gomez Perdiguero et al., 2015; Hoeffel et al., 2015) and CD45^+^ CD41^+^ KIT^+^ EMPs (McGrath et al., 2015; Frame et al., 2016), as well as lymphomyeloid progenitors (Boiers et al., 2013). The smaller proportion of KIT^+^ CD41^low^ progenitors that did not express CD45 (Figure 1E) may include EMPs that are CD45^–^ (McGrath et al., 2015), possibly because they are less mature, as well as other KIT^+^ CD41^+^ CD45^–^ hematopoietic precursors that have recently been described (Yamane, 2018).

**FIGURE 2:**
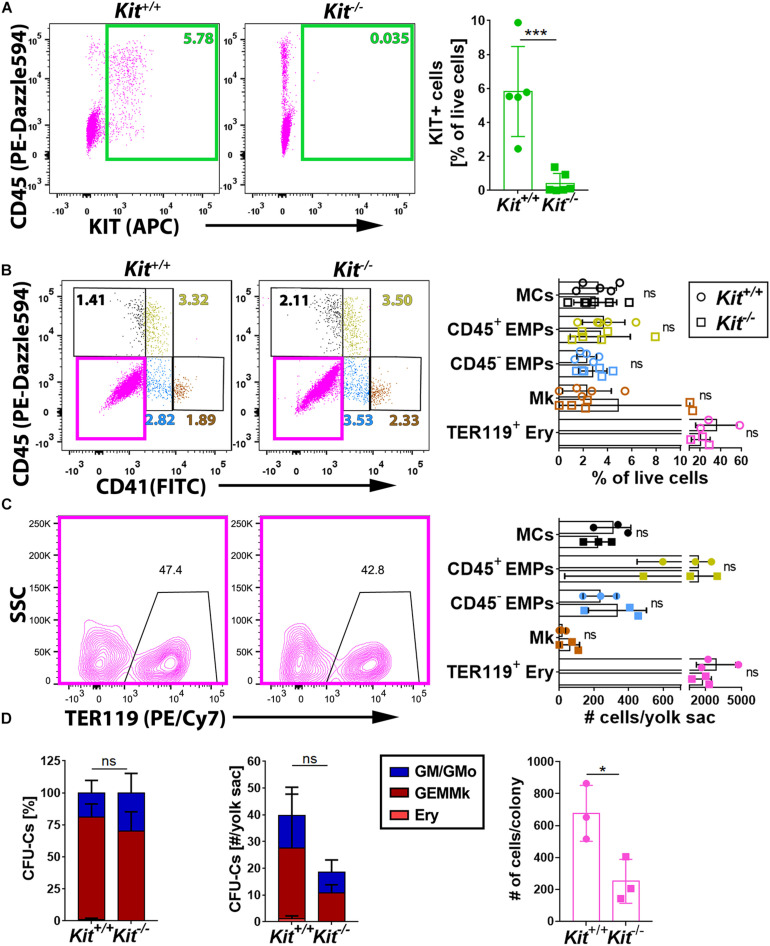
KIT is dispensable for yolk sac hematopoiesis. **(A)** Flow cytometry analysis of single living cells from E9.5 *Kit*^+/+^ and *Kit^–/–^* littermate mouse yolk sacs (*n* = 5 each) with the indicated markers. The green boxes in the scatter plots indicate the gates used to determine the percentage of KIT^+^ cells. Data are shown as mean ± SD; each data point represents the value from one embryo; ***p < 0.001 (unpaired Student’s *t*-test). **(B,C)** Flow cytometry analysis of single living cells from E9.5 *Kit*^+/+^ and *Kit^–/–^* (*n* ≥ 3 each) littermate mouse yolk sacs with the indicated markers. The color-coded boxes or annotation in the scatter plots indicate the gates used to determine the percentage (top bar graph) as well as the embryo equivalent number (bottom bar graph) of cell populations defined by CD45, CD41, or TER119 expression levels. Data are shown as mean ± SD; each data point represents the value from one embryo; non-significant (two-way ANOVA followed by Sidak’s multiple comparisons test). **(D)** Quantification of the percentage (left panel), number (center panel), and colony cell number of CFU-C (right panel) in *Kit*^+/+^ versus *Kit^–/–^* littermate E9.5 yolk sacs (*n* = 3 each). Each data point represents the value from one embryo; stacked bar graph data are shown as mean ± SEM; ns, non-significant (two-way ANOVA); bar graph data are shown as mean ± SD; *p < 0.05 (unpaired Student’s *t*-test).

To understand whether KIT promotes EMP formation and/or affects the differentiation potential of EMPs, we performed flow cytometry analysis of yolk sacs from mice lacking KIT. For this experiment, we used mice homozygous for the *Kit*^*CreERT2*^ knock-in allele, in which the *Cre* recombinase gene is inserted into the endogenous *Kit* locus (Klein et al., 2013) to generate a true *Kit* null allele (Heger et al., 2014). These mice are hereafter referred to as *Kit^–/–^* mutants. Notably, KIT loss in E9.5 yolk sacs (Figure 2A) did not significantly alter the proportion or the number of the CD45^–^ CD41^–^ TER119^+^ erythroblasts (Frame et al., 2016), the CD45^–^ CD41^+^ population that includes megakaryoblasts, the CD41^low^ progenitor population that includes both CD45^+^ and CD45^–^ EMPs, or the CD45^+^ CD41^–^ population corresponding to differentiating MCs (Figures 2B,C). Moreover, functional CFU-C assays with E9.5 yolk sac cells identified progenitors with either mixed erythro-myeloid (GEMMk: granulocyte, erythroid, monocyte/macrophage, megakaryocyte) or a more myeloid committed potential (GM: granulocyte, monocyte/macrophage; GMo: granulocyte, monocyte) in similar proportions in KIT-deficient and littermate control embryos (Figure 2D and Supplementary Figure 1). By contrast, the number of functional progenitors in the mutants appeared reduced, albeit not at statistically significant levels (Figure 2D). Moreover, the colonies that grew from the mutant progenitors contained significantly fewer cells than those of wild-type littermates (Figure 2D), which indicates that the progenitors have reduced proliferative capacity.

Taken together, the analysis of KIT-deficient mice at E9.5 suggests that KIT is dispensable for primitive yolk sac hematopoiesis and for EMP formation from hemogenic endothelium in the yolk sac, but that it is required cell autonomously in EMPs for their expansion.

### KIT Is Dispensable for Yolk Sac-Derived Macrophage Colonization of Embryonic Organs

**FIGURE 3:**
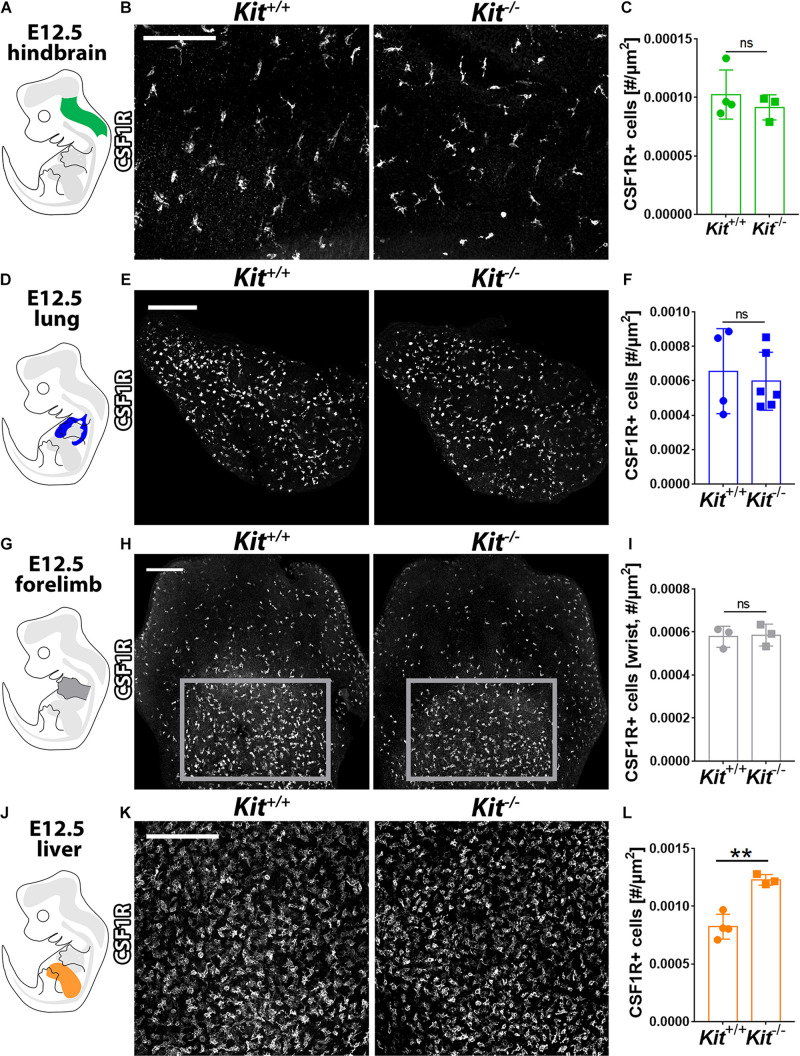
KIT loss does not affect yolk sac-derived macrophage colonization of embryonic brain, lung, and limb but promotes their expansion in the liver. E12.5 mouse hindbrain **(A–C)**, lung **(D–F)**, forelimb **(G–I)**, and liver **(J–L)** from *Kit*^+/+^ versus *Kit^–/–^* littermates were whole mount stained for CSF1R, imaged by confocal microscopy, and shown in gray scale. Scale bars: 200 μm. In **(H)**, the areas indicated with gray rectangles indicate the regions used for the quantification of CSF1R + macrophages in the wrist area of the forelimb. Quantification of CSF1R + macrophages: *n* = 4 wild-type and 3 mutant hindbrains from three litters **(C)**, *n* = 4 wild-type and 6 mutant lungs from three litters **(F)**, *n* = 3 each for forelimbs from two litters **(I)**, and *n* = 4 wild-type and 3 mutant livers from three litters **(L)**. Bar graph data are shown as mean ± SD; each data point represents the value from one embryo; ns, non-significant; **p < 0.01 (unpaired Student’s *t*-test).

Yolk sac-born macrophages give rise to microglia (Schulz et al., 2012; Kierdorf et al., 2013; Gomez Perdiguero et al., 2015; Hoeffel et al., 2015). They also contribute to the initial pool of tissue-resident macrophages in several other developing organs, including lung, liver, and skin, where they constitute the main macrophage population at E12.5 (Hoeffel et al., 2015) before being complemented by monocytes derived from liver EMPs (Ginhoux and Guilliams, 2016). These yolk sac-derived tissue macrophages can be distinguished by high F4/80 and CSF1R expression from the monocyte-derived macrophages derived from liver EMPs, which have low F4/80 levels (Schulz et al., 2012; Hoeffel et al., 2015). CSF1R immunofluorescence analysis showed that the number of microglia was similar between *Kit*^+/+^ and *Kit^–/–^* hindbrains at E12.5 (Figures 3A–C). Moreover, CSF1R immunostaining showed a similar number of tissue macrophages in the E12.5 lung and forelimb of *Kit*-null embryos and their wild-type littermates (Figures 3D–I). By contrast, the number of tissue macrophages in the E12.5 liver (termed Kupffer cells) appeared significantly increased in mutants compared to wild-type controls (Figures 3J–L). KIT is therefore not required for the formation of yolk sac-derived macrophages (Figure 2) or their colonization of embryonic organs (Figure 3).

### KIT Is Required for Transient-Definitive Fetal Liver Hematopoiesis

**FIGURE 4:**
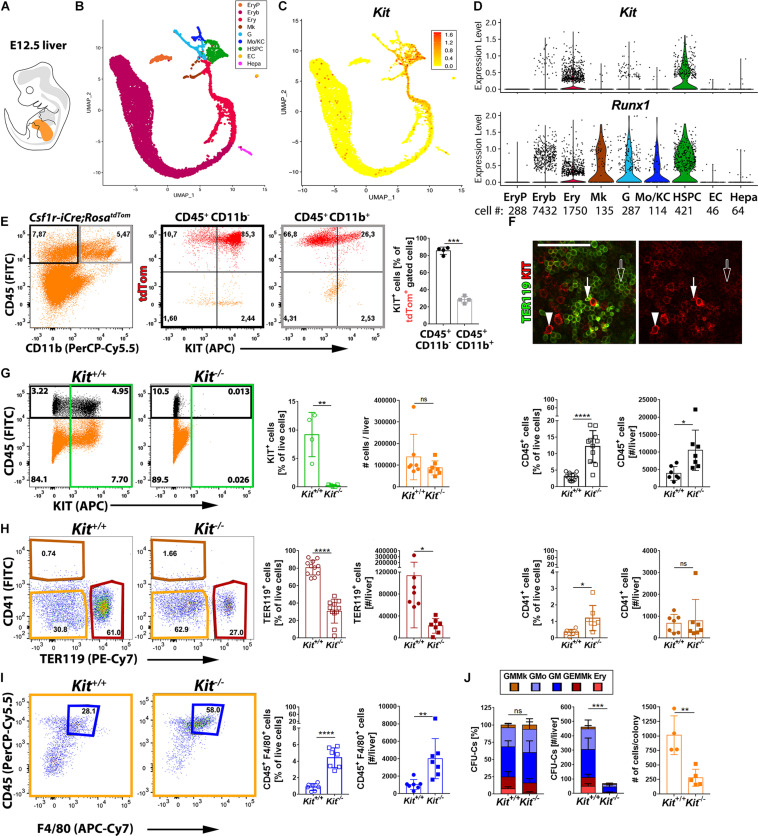
*Kit* expression profiling and requirement during fetal liver hematopoiesis. **(A)** Schematic representation of an E12.5 mouse embryo with the fetal liver shown in orange. **(B–D)** scRNA-seq analysis of E12.5 mouse liver. UMAP plots were used to visualize clusters of distinct cell types **(B)** and *Kit* transcript levels in each cell **(C)**. Violin plots **(D)** illustrate *Kit* and *Runx1* single-cell transcript levels in each cluster; the number of cells in each cluster is indicated below the violin plots. **(E)** Flow cytometry analysis of single living cells from E12.5 *Csf1r-iCre;Rosa^*tdTom*^* fetal mouse livers with the indicated markers. The boxes in the left-hand scatter plot indicate the gates used to generate the scatter plots of cells expressing CD45 only (black frame) or CD45 and CD11b (gray frame). The bar graph illustrates that the proportion of KIT^+^ tdTomato^+^ cells is enriched in the CD45^+^ CD11b^–^ compared to the CD45^+^ CD11b^+^ population (*n* = 4 livers). Data are shown as mean ± SD; each data point represents the value from one liver. ***p < 0.001 (paired Student’s *t*-test). **(F)** Whole-mount labeling of E12.5 mouse liver with the indicated markers (*n* = 2). White arrowheads indicate KIT^+^ TER119^–^ hematopoietic progenitors, arrows denote KIT^+^ TER119^+^ proerythroblasts, and empty arrows indicate KIT^–^ TER119^+^ erythroblasts; scale bar: 100 μm. **(G–I)** Flow cytometry analysis of single living cells from E12.5 *Kit^–/–^* and *Kit*^+/+^ littermate mouse livers with the indicated markers. The color-coded boxes in the scatter plots indicate the gates used to analyze specific cell populations, shown in corresponding colors in the bar charts. Data are shown as mean ± SD; each data point represents the value from one liver; ns, non-significant, *p < 0.05, **p < 0.01, ****p < 0.0001 (unpaired Student’s *t*-test). **(G)** Loss of KIT^+^ cells in *Kit*-null mutants (green; *n* = 4 wild types and *n* = 5 mutants), total cell number (orange: *n* = 7 each) and percentage as well as the number of CD45^+^ cells in each liver (black; *n* = 11 and *n* = 7 each, respectively). **(H,I)** Quantification of percentage as well as the number of TER119^+^ CD41^–^ erythroid cells (red; *n* = 11 and *n* = 7 each, respectively) and CD41^+^ TER119^–^ megakaryocytes (brown; *n* = 7 each). The double-negative population (orange) was further analyzed to identify CD45^+^ F4/80^+^ macrophages (blue; *n* = 7 each). **(J)** Quantification of the percentage (left panel), number (center panel), and colony cell number (right panel) of CFU-C in *Kit*^+/+^ versus *Kit^–/–^* littermate E12.5 livers (*n* = 4 wild types and *n* = 5 mutants). Each data point represents the value from one embryo; stacked bar graph data are shown as mean ± SEM; ns, non-significant; ***p < 0.001 (two-way ANOVA); bar graph data are shown as mean ± SD; **p < 0.01 (unpaired Student’s *t*-test).

Starting from E10.5 onward, EMPs leave the yolk sac and colonize the fetal liver (Lux et al., 2008; Frame et al., 2013; Hoeffel et al., 2015; McGrath et al., 2015; Hoeffel and Ginhoux, 2018). To define the KIT^+^ cell populations in the fetal liver, we analyzed the E12.5 mouse liver transcriptome by scRNA-seq (detailed characterization in a manuscript in preparation). Consistent with the liver being the major hematopoietic organ at E12.5, our annotation identified several hematopoietic cell types in a UMAP continuum. Thus, a cluster of hematopoietic stem and progenitor cells (HSPCs, *Myb*^+^), composed mostly of EMPs and also the first HSCs, branched into several cell trajectories defined by markers of granulocytes (G, *Ly6g*^+^), monocytes and Kupffer cells (Mo/KC, *Fcgr1*^+^), megakaryocytes (Mk, *Pf4*^+^), or erythroid-committed progenitors such as burst-forming unit erythroid (BFU-E) cells (Ery, *Klf1*^+^ *Rhd*^–^) and erythroblasts (Eryb, *Klf1*^+^ *Rhd*^+^) (Figures 4A,B). As expected, we also identified distinct clusters of yolk sac-derived primitive orthochromatic erythroblasts (EryP, *Hba-x*^+^) as well as hepatoblasts (Hepa, *Alb*^+^) and ECs (*Cldn5*^+^) (Figure 4B).

We next examined which liver cell types expressed *Kit*. Hepatoblasts and fetal liver ECs did not contain *Kit* transcripts or the hemogenic endothelium marker *Runx1* (Figures 4C,D). These findings support the idea that the fetal liver, even though it harbors recruited hematopoietic progenitors (Lux et al., 2008; Frame et al., 2013; Hoeffel et al., 2015; McGrath et al., 2015; Hoeffel and Ginhoux, 2018), lacks hemogenic endothelium. *Kit* transcripts were also not detected in primitive erythroblasts, differentiating megakaryocytes, or differentiated MCs (Figures 4C,D). By contrast, *Kit* transcripts were abundant in the HSPC population that includes EMPs (Figures 4C,D). *Kit* transcripts were also abundant in erythroid-committed progenitors (BFU-E), but the number of *Kit*^+^ cells gradually decreased in erythroid-committed progenitors as they differentiated toward an erythroblast phenotype, with barely any *Kit*^+^ cells present in the erythroblast cluster (Figures 4C,D).

Liver EMPs can also be identified by flow cytometry in the E12.5 *Csf1r-iCre;Rosa^*tdTom*^* liver as CD45^+^ tdTomato^+^ cells lacking the differentiated MC marker CD11b. In agreement with the scRNA-seq data, we found that ∼90% of EMPs in wild-type liver were KIT^+^ (Figure 4E). In contrast, CD45^+^ tdTomato^+^ CD11b^+^ cells mostly lacked KIT (Figure 4E), agreeing with the finding that *Kit* transcripts were not identified in differentiated MCs (Figures 4C,D). Moreover, KIT^+^ cells were also detected in the CD45^–^ population, both within the TER119^+^ erythroid cells and the TER119^–^ cells that likely include erythroid progenitors (Supplementary Figure 2). Immunostaining confirmed that KIT is expressed in large and round TER119^–^ cells that likely represent hematopoietic progenitors and only a few of the smaller TER119^+^ erythroblasts (Figure 4F), most likely proerythroblasts (Azzoni et al., 2018). This expression pattern is compatible with a selective role for KIT in hematopoietic progenitor populations that include EMPs and their erythroid-committed progeny during fetal liver erythropoiesis.

We next examined how KIT loss affected hematopoietic cells including EMP progeny in the liver (Figures 4G–I). Overall, organ cellularity was similar in the liver of E12.5 KIT-deficient and wild-type mice (Figure 4G). Nevertheless, both the proportion and total number of TER119^+^ cells were significantly decreased in mutants (Figure 4H). By contrast, both the proportion and total number of CD45^+^ cells were significantly increased (Figure 4G). Additionally, the proportion of CD41^+^ megakaryocytes (Figure 4H) and both the proportion and total number of CD45^+^ F4/80^+^ macrophages (Figure 4I) were significantly increased. The increase in non-erythroid hematopoietic cells may explain why organ cellularity was not decreased in KIT-deficient livers, despite impaired erythropoiesis.

To better understand the origin of this hematopoietic imbalance, we compared the clonogenic potential of hematopoietic progenitors in the E12.5 liver from KIT-deficient and littermate control embryos. In both genotypes, we observed similar proportions of mixed erythro-myeloid progenitors (GEMMk) and progenitors with a more lineage-committed potential (GM; GMo; GMMk: granulocyte, monocyte/macrophage, megakaryocyte; Ery: erythroid) (Figure 4J and Supplementary Figure 1). Finding that the clonogenic potential of liver EMPs is preserved despite KIT deficiency implies that KIT does not play a role in fate decisions made by EMPs as they differentiate into committed progenitors in the fetal liver. Nevertheless, CFU-C assays revealed a significant decrease in the absolute number of clonogenic progenitors across all lineages in *Kit*-null livers, with virtually no erythroid or mixed erythro-myeloid colonies (Figure 4J). Moreover, the colonies produced by the remaining progenitors from mutant livers contained significantly fewer cells (Figure 4J). These results identify an essential role for KIT in progenitor expansion after their recruitment into the liver.

## Discussion

In recent years, it has become clear that EMPs form in the yolk sac but, starting from E10.5, they migrate from the yolk sac into the fetal liver (Palis and Yoder, 2001), where they expand and differentiate (Ginhoux and Guilliams, 2016). Our analysis showed KIT localization to a cluster of hematopoietic progenitors that appeared to bud from yolk sac ECs at E9.5 and therefore likely represent EMPs (Figure 1). Contrary to prior *ex vivo* findings, which concluded that KIT is required for hemogenic endothelial cell (EC) specification and function *ex vivo* (Marcelo et al., 2013), our flow cytometry analysis of KIT null mutants showed that KIT is dispensable for the generation of CD41^low^ progenitors, which include both CD45^+^ and CD45^–^ KIT^+^ EMPs (Figure 2). Furthermore, we found that KIT is dispensable for the formation of differentiated hematopoietic cells in the E9.5 yolk sac (Figure 2), which are generated by both primitive progenitors and the earliest wave of EMPs (Hoeffel and Ginhoux, 2018). These findings, in turn, suggest that hemogenic endothelium can form and function even in the absence of KIT to generate EMPs.

EMPs arising in E8.5 and E9.5 yolk sacs exhibit erythroid and broad myeloid, but not lymphoid potential (Frame et al., 2013; Ginhoux and Guilliams, 2016). Notably, we observed a diminished proliferative capacity for E9.5 yolk sac EMPs in KIT-deficient mice, together with a trend for fewer clonogenic progenitors overall (Figure 2). This impairment became statistically significant in fetal liver of the E12.5 KIT-deficient mice, which showed a major decrease in the number of functional hematopoietic progenitors as well as their proliferative capacity (Figure 4). Our observations agree with a recent study of mice lacking the KIT ligand KITL (also known as stem cell factor, SCF), which is expressed by yolk sac and fetal liver ECs as well as fetal liver stromal cells (Azzoni et al., 2018). Specifically, this prior study reported the normal emergence of EMPs in the yolk sac at E9.5 but a striking decrease in their number in the fetal liver (Azzoni et al., 2018). This reduction in liver EMPs was ascribed to reduced EMP proliferation in the yolk sac after E9.5, which resulted in a decreased number of circulating EMPs and reduced their colonization, proliferation, and survival in the liver (Azzoni et al., 2018).

Our single-cell transcriptomic analysis of E12.5 fetal liver demonstrated that KIT transcripts are not detectable in ECs at this stage (Figure 4). Lack or low levels of *Kit* expression in E12.5 liver ECs is unexpected, because *Kit* is abundantly expressed in adult hepatic sinusoidal ECs (Mansuroglu et al., 2009); see also Tabula Muris database^1^ (Tabula Muris Consortium et al., 2018). Our findings therefore suggest that robust *Kit* expression in liver ECs is acquired later on during development, perhaps after the liver vasculature has specialized into the sinusoids that connect the portal triads to the central veins (Strauss et al., 2017). Instead, KIT transcripts were enriched in liver hematopoietic progenitors, including EMPs and erythroid progenitors such as the BFU-E (Figure 4). This expression pattern agrees with the finding that KIT is required for EMP expansion, which starts in the E9.5 yolk sac downstream of EMP formation from the yolk sac hemogenic endothelium (Figure 2) and continues in the E12.5 liver (Figure 4). Moreover, *Kit* expression in liver erythroid progenitors is consistent with a KIT requirement for EMP-dependent transient-definitive erythropoiesis taking place in the fetal liver (Figure 4), a process that is required to sustain embryonic life until birth (Soares-da-Silva et al., 2021). Our study therefore provides new insights into the precise requirement of KIT in specific progenitor populations for developmental erythropoiesis. Specifically, our study refines previous work based on *Kit*^*W/W*^ and *Kitl*^*Sl/Sl*^ spontaneous mutants (Russel et al., 1968; Chui and Russell, 1974; Chui et al., 1978) and neutralizing anti-KIT antibodies (Ogawa et al., 1993), which showed an erythroid differentiation block in the fetal liver after E13.5 without identifying the specific progenitor population involved. Accordingly, we have identified EMP expansion and erythroid differentiation from EMP-derived progenitors in the fetal liver as the first events in which KIT acts to prevent severe anemia.

We further show that KIT is dispensable for the generation of tissue-resident macrophages in the E12.5 brain, lung, and limb bud (Figure 3). This finding agrees with KIT being dispensable for the generation of MCs in the yolk sac (Figure 2), because tissue macrophages in early embryonic organs are known to differentiate from hematopoietic progenitors that arise in the yolk sac before the birth of the liver-colonizing EMPs (Ginhoux and Guilliams, 2016; Hoeffel and Ginhoux, 2018). Our observations also agree with findings in mice lacking KITL (Azzoni et al., 2018), in which the proportion of yolk sac-derived tissue macrophages (F4/80^hi^ CD11b^lo^) in the brain, lungs, and limb buds are unaffected at E14.5 (Azzoni et al., 2018). However, we have also observed a notable difference between the analyses of KIT- and KITL-deficient mice. Thus, KIT-deficient mice have an increased population of yolk sac-derived macrophages in the E12.5 liver compared to wild-type littermates (Figure 3), but the liver of E11.5 or E14.5 KITL-deficient mice does not (Azzoni et al., 2018). It is not known whether this difference in ligand and receptor mutant mice is due to the different embryonic stages examined (E12.5 vs. 11.5 and E14.5) or whether defective liver hematopoiesis caused by KIT deficiency indirectly allows for early tissue macrophage expansion, whereas KITL deficiency does not.

Notably, the population of F4/80^lo^ CD11b^hi^ monocyte-derived macrophages, which are derived from fetal liver EMPs, is significantly reduced in the E14.5 liver lacking KITL (Azzoni et al., 2018). This observation is consistent with the reduced number of clonogenic fetal liver progenitors across all lineages and their reduced expansion, a phenotype that is observed for both E11.5 KITL-deficient mice (Azzoni et al., 2018) and E12.5 *Kit*-null mice (Figure 4). While we have not specifically examined whether the population of E14.5 monocyte-derived macrophages is affected downstream of the E12.5 EMP defect in *Kit*-null mice, it stands to reason that the overall increase in macrophage numbers in the liver of *Kit* mutants at E12.5 might be explained by a KIT deficiency-induced mechanism that promotes the self-renewal of yolk sac-derived macrophages to compensate for the reduction in liver EMP and EMP-derived cells, which are mostly erythroblasts at this stage.

The central finding of our study is the KIT requirement downstream of EMP formation in the yolk sac, with a key role in EMP expansion and regulating transient-definitive fetal liver hematopoiesis across several cell lineages to affect erythroid, myeloid, and megakaryocyte generation (see working model, Supplementary Figure 3). At E12.5, KIT loss has a major impact on fetal liver erythropoiesis, which normally peaks at E12.5 (McGrath et al., 2011; Iturri et al., 2021). By contrast, the reduction in myeloid output from liver EMPs in the mutants cannot yet be appreciated at E12.5, when the tissue macrophage population is still mostly of yolk sac origin (Hoeffel et al., 2015). Moreover, the local expansion of the yolk sac-derived macrophage population (Figure 3) would likely mask any deficiency in myeloid output from liver EMPs (Supplementary Figure 3, model). In agreement with our observation that KIT promotes erythroid development, gain-of-function mutations in the *Kit* coding sequence have been described to trigger clonal expansion of malignant pro-erythroblasts in murine erythroleukemia (Kosmider et al., 2005). Moreover, compatible with KIT regulating the expansion of EMPs with their intrinsic myeloid potential, gain-of-function mutations have been associated with human adult and pediatric core binding factor acute myeloid leukemia (CBF-AML), for which KIT mutations are poor prognostic factors (Cairoli et al., 2006; Krauth et al., 2014; Chen et al., 2018). It should therefore be investigated whether *KIT* mutations also contribute to human erythroleukemia.

## Methods

See Supplementary Material.

## Data Availability Statement

Fetal liver scRNAseq data can be found here: [https://www.ncbi.nlm.nih.gov/geo/, GSE180050]. The original contributions presented in the study are included in the article/Supplementary Material, further inquiries can be directed to the corresponding authors.

## Ethics Statement

The animal study was reviewed and approved by the Animal Welfare Ethical Review Body (AWERB) and UK Home Office.

## Author Contributions

AF and CR contributed to the conception, design of the study, and co-wrote the manuscript. AF, CT, CR, and LD performed mouse experiments. AF and CT analyzed data. EV and EC performed bioinformatic analyses. All authors read and approved the submitted manuscript.

## Conflict of Interest

The authors declare that the research was conducted in the absence of any commercial or financial relationships that could be construed as a potential conflict of interest.

## Publisher’s Note

All claims expressed in this article are solely those of the authors and do not necessarily represent those of their affiliated organizations, or those of the publisher, the editors and the reviewers. Any product that may be evaluated in this article, or claim that may be made by its manufacturer, is not guaranteed or endorsed by the publisher.

## Footnotes

1
https://tabula-muris.ds.czbiohub.org/
