# Supplementary material for: KIT Is Required for Fetal Liver Hematopoiesis
Source: Front Cell Dev Biol. 2021 Jul 29;9:648630. doi: 10.3389/fcell.2021.648630 (PMC8358609; doi:10.3389/fcell.2021.648630)
Supplement: Supplementary file 1 [file Data_Sheet_1.PDF]

Methods and Supplementary Figures

Methods

Mouse strains

All animal procedures were performed in accordance with the institutional Animal Welfare Ethical Review Body (AWERB) and UK Home Office guidelines. To obtain mouse embryos of defined gestational age, mice were paired in the evening and the presence of a vaginal plug the following morning was defined as E0.5. Mice carrying the *Csf1r-iCre* transgene (Deng et al., 2010) were mated to mice carrying the *Cre* recombination reporters $Rosa^{Yfp}$ (Srinivas et al., 2001) or $Rosa^{tdTom}$ (Madisen et al., 2010). Embryos lacking *Kit* expression were generated by mating $Kit^{CreERT2}$ mice (Klein et al., 2013) or by mating $Kit^{CreERT2}$ mice carrying $Rosa^{tdTom}$ and *Csf1r-iCre* to obtain *Csf1r-iCre;*$Rosa^{tdTom}$;$Kit^{-/-}$ embryos. All mouse strains were maintained on a mixed background (C57Bl6/J;129/Sv, C57Bl6/J;FVB or C57Bl6/J;129/Sv;FVB).

Wholemount tissue staining

Samples were fixed in 4% formaldehyde in PBS and processed as wholemounts for fluorescent staining as described previously for wholemount hindbrains (Fantin et al., 2013). We used the following antibodies and dilutions: rat anti-CDH5 (1:200; 555289, BD Pharmingen), rat anti-KIT (1:500; 553353, lot 30259, BD Pharmingen), rat anti-KIT APC-conjugated (1:200; clone 2B8, cat 105812, lot B217855, Biolegend), rabbit anti-RFP (1:1,000; PM005, lot 045, MBL), rabbit anti-CSF1R (1:500; sc-692, lot K1212, Santa Cruz Technologies), goat anti-VEGFR2 (1:200; AF644, lot COA0417021, R&D Systems), goat anti-CDH5 (1:200; AF1002, lot FQI0116101, R&D Systems), rat anti-TER-119 (1:200, clone TER-119, cat 116222, Biolegend). Secondary antibodies used included donkey fluorophore-conjugated FAB fragments of anti-goat, -rabbit or -rat IgG (Jackson ImmunoResearch). Images were acquired with a LSM710 (Zeiss) or an A1 (Nikon) laser scanning confocal microscope and processed using LSM image browser (Zeiss), Fiji (NIH Bethesda)(Schindelin et al., 2012) and Photoshop CS4 (Adobe Inc.) software. Three-dimensional reconstructions including surface rendering for channel masking were performed using Imaris (Bitplane). *Z*-stack projections of confocal images are shown unless indicated otherwise in the figure legends.

Flow cytometry

Tissues were mechanically and enzymatically homogenized in RPMI1640 with 2.5% fetal bovine serum (FBS, ThermoFisher), 100 μg/ml collagenase/dispase (Roche), 50 μg/ml DNase (Qiagen) and 100 μg/ml heparin (Sigma), incubated for 15 min with 0.5 mg/ml rat Fc block (Becton Dickinson) and labelled with a combination of BV585-, PerCp-Cy5.5-, PE-Dazzle 594- or FITC-conjugated rat anti-CD45 (clone 30-F11) or FITC-conjugated rat anti-CD41 (clone MWReg30, cat 133903, lot B201955),

PerCp-Cy5.5- or APC-conjugated rat anti-KIT (clone 2B8), PE-Cy7- or FITC-conjugated rat anti-TER-119 (clone TER-119), PerCp-Cy5.5-conjugated rat anti-CD11b (clone M1/70, cat 101227), APC-Cy7-conjugated rat anti-F4/80 (clone BM8, cat 123118) (all BioLegend) or AF647-conjugated rat anti-F4/80 (clone Cl:A3-1, BioRad). Appropriate fluorescence gating parameters were established with unstained or *Csf1r-iCre*-negative tissues and fluorescence-minus-one (FMO) staining. In all experiments, doublets were eliminated using pulse geometry gates (FSC-H versus FSC-A and SSC-H versus SSC-A), whereas dead cells were removed using SYTOX Blue, DAPI or LIVE/DEAD Fixable eFluor450 (all Life Technologies). Single-cell suspensions were analyzed using the BD LSRFortessa X-20 cell analyser or sorted using the BD Influx cell sorter or FACSAria II (BD Biosciences); FlowJo v10.6.2 software (Tree Star/FlowJo LLC) was used for subsequent analyses. In experiments for absolute cell number quantification, tissues were stored at -80°C in FBS containing 10% dimethyl sulfoxide (DMSO) prior to dissociation.

Colony-forming unit cell (CFU-C) assays

Yolk sacs and livers were stored at -80°C in FBS containing 10% DMSO before they were mechanically and enzymatically digested in DMEM with 2% fetal FBS (ThermoFisher), 1.2 mM $CaCl_2$, 1 mg/ml collagenase/dispase (Roche), 40 µg/ml DNase (Roche) and 100 µg/ml heparin (Sigma) on a thermoshaker at 37°C for 20 min. Afterwards, homogenized samples were filtered with a 40-µm cell strainer (Greiner) and washed with PBS, 2% FBS and PenStrep (Sigma), centrifuged and counted. Cells were then plated in Methocult (StemCell Technologies) as previously described (Azzoni et al., 2018). Briefly, 4500 cells from yolk sac and 15000 cells from liver were resuspended in 200 µl of PBS, 2% FBS and PenStrep, mixed with 2.8 ml of M3434 (StemCell Technologies) and 1 ml of cell mixture was plated in duplicate in a 6 cm low-adherence dish (Greiner) and cultured at 37°C, 5% CO_2. Colonies were scored after 11 days by visual inspection and flow cytometry. Single colonies were aspirated, washed and incubated for 15 min with rat Fc block (1:100, 101302, Biolegend) and labelled with AF647-conjugated rat anti-F4/80 (1:200, clone Cl:A3-1, cat MCA497A647, AbD Serotec), PerCp-Cy5.5-conjugated rat anti-CD45 (1:200, clone 30-F11, cat 103132, Biolegend), FITC-conjugated rat anti-CD41 (1:200, clone MWReg30, cat 133903, Biolegend), APC-Cy7-conjugated GR1 (1:200, clone RB6-8C5, cat 108423, Biolegend) and PE-Cy7-conjugated rat anti-TER119 (1:200, clone TER-119, cat 116221, Biolegend). After washing, cells were resuspended in FACS buffer for acquisition. Single-cell suspensions were analyzed using the FACSAria II (BD Biosciences); FlowJo v10.6.2 software (Tree Star/FlowJo LLC) was used for subsequent analyses.

Single cell RNA sequencing (scRNA-seq) analysis

The E12.5 liver scRNA-seq dataset was generated from C57BL/6J fetal liver (manuscript in preparation). Briefly, a single cell suspension from one E12.5 liver, obtained by enzymatic digestion and mechanical dissociation was purified from debris, dead cells and doublets through fluorescence activated cell sorting (FACS). To maximise the yield in droplet encapsulation of single cells, two

separate technical replicates of the sample were independently processed. Next, the library was prepared using the Chromium Single-Cell Controller (10X Genomics, Pleasanton, CA) and sequenced at a depth of 50k reads/cell on a NovaSeq 6000 (Illumina). Raw files were processed with Cell Ranger 2.1.1 and reads mapped to the mm10 genome and counted with GRCm38.92 annotation. Using RStudio 3.6.3 and the R package Seurat 3.2.0, cells containing feature counts less than 200 feature counts and genes detected in less than 3 cells were removed, obtaining 5,480 and 5,057 cells, respectively, for the two technical replicates, which were then merged into a single sample for subsequent analysis. Downstream analysis included data normalization ("LogNormalize" method and scale factor of 10,000) and variable gene detection ("vst" selection method, returning 2,000 features per dataset). The principal components analysis (PCA) was performed on variable genes, and the optimal number of principal components, PCs, for each sample was chosen using the elbow plot (15). Louvain graph-based clustering was performed using the selected PCs and a resolution of 0.6. Dimensionality reduction methods included UMAP, and cluster cell identity was assigned by manual annotation using known marker genes.

Statistical analysis

Tissues for analysis were allocated to experimental groups according to genotype, gestational age, organ or cell type, rather than being randomized. To ensure the unbiased interpretation of results, the genotype and gestational age were disclosed only after data collection was complete, but the investigators knew the sample origin (i.e., organ or cell type). No statistical methods were used to predetermine sample size. For E9.5 yolk sac image analysis, the mean gray value for CDH5 immunostaining was quantified in the cytoplasm of KIT^{+} EMPs and KIT^{-} ECs using Fiji (Schindelin et al., 2012). The number of macrophages in the hindbrain, lung (whole left lobe), forelimb and liver were quantified with Fiji. For all experiments, the error bars represent the standard deviation of the mean (unless otherwise stated, see legends). Comparison of medians against means justified the use of a parametric test; to determine whether two datasets were significantly different, we therefore calculated P values with a two-tailed unpaired Student's *t*-test; $P < 0.05$ was considered significant. Statistical analyses were performed with Excel 12.2.6 (Microsoft Office) or Prism 7 (GraphPad Software).

References

Azzoni, E., V. Frontera, K.E. McGrath, J. Harman, J. Carrelha, C. Nerlov, J. Palis, S.E.W. Jacobsen, and M.F. de Bruijn. 2018. Kit ligand has a critical role in mouse yolk sac and aorta-gonad-mesonephros hematopoiesis. *EMBO Rep*. 19.

Deng, L., J.F. Zhou, R.S. Sellers, J.F. Li, A.V. Nguyen, Y. Wang, A. Orlofsky, Q. Liu, D.A. Hume, J.W. Pollard, L. Augenlicht, and E.Y. Lin. 2010. A novel mouse model of inflammatory bowel disease links mammalian target of rapamycin-dependent hyperproliferation of colonic

epithelium to inflammation-associated tumorigenesis. *The American journal of pathology*. 176:952-967.

Fantin, A., J.M. Vieira, A. Plein, C.H. Maden, and C. Ruhrberg. 2013. The embryonic mouse hindbrain as a qualitative and quantitative model for studying the molecular and cellular mechanisms of angiogenesis. *Nature protocols*. 8:418-429.

Klein, S., B. Seidler, A. Kettenberger, A. Sibaev, M. Rohn, R. Feil, H.D. Allescher, J.M. Vanderwinden, F. Hofmann, M. Schemann, R. Rad, M.A. Storr, R.M. Schmid, G. Schneider, and D. Saur. 2013. Interstitial cells of Cajal integrate excitatory and inhibitory neurotransmission with intestinal slow-wave activity. *Nature communications*. 4:1630.

Madisen, L., T.A. Zwingman, S.M. Sunkin, S.W. Oh, H.A. Zariwala, H. Gu, L.L. Ng, R.D. Palmiter, M.J. Hawrylycz, A.R. Jones, E.S. Lein, and H. Zeng. 2010. A robust and high-throughput Cre reporting and characterization system for the whole mouse brain. *Nature neuroscience*. 13:133-140.

Schindelin, J., I. Arganda-Carreras, E. Frise, V. Kaynig, M. Longair, T. Pietzsch, S. Preibisch, C. Rueden, S. Saalfeld, B. Schmid, J.Y. Tinevez, D.J. White, V. Hartenstein, K. Eliceiri, P. Tomancak, and A. Cardona. 2012. Fiji: an open-source platform for biological-image analysis. *Nature methods*. 9:676-682.

Srinivas, S., T. Watanabe, C.S. Lin, C.M. William, Y. Tanabe, T.M. Jessell, and F. Costantini. 2001. Cre reporter strains produced by targeted insertion of EYFP and ECFP into the ROSA26 locus. *BMC developmental biology*. 1:4.

Supplementary figure 1

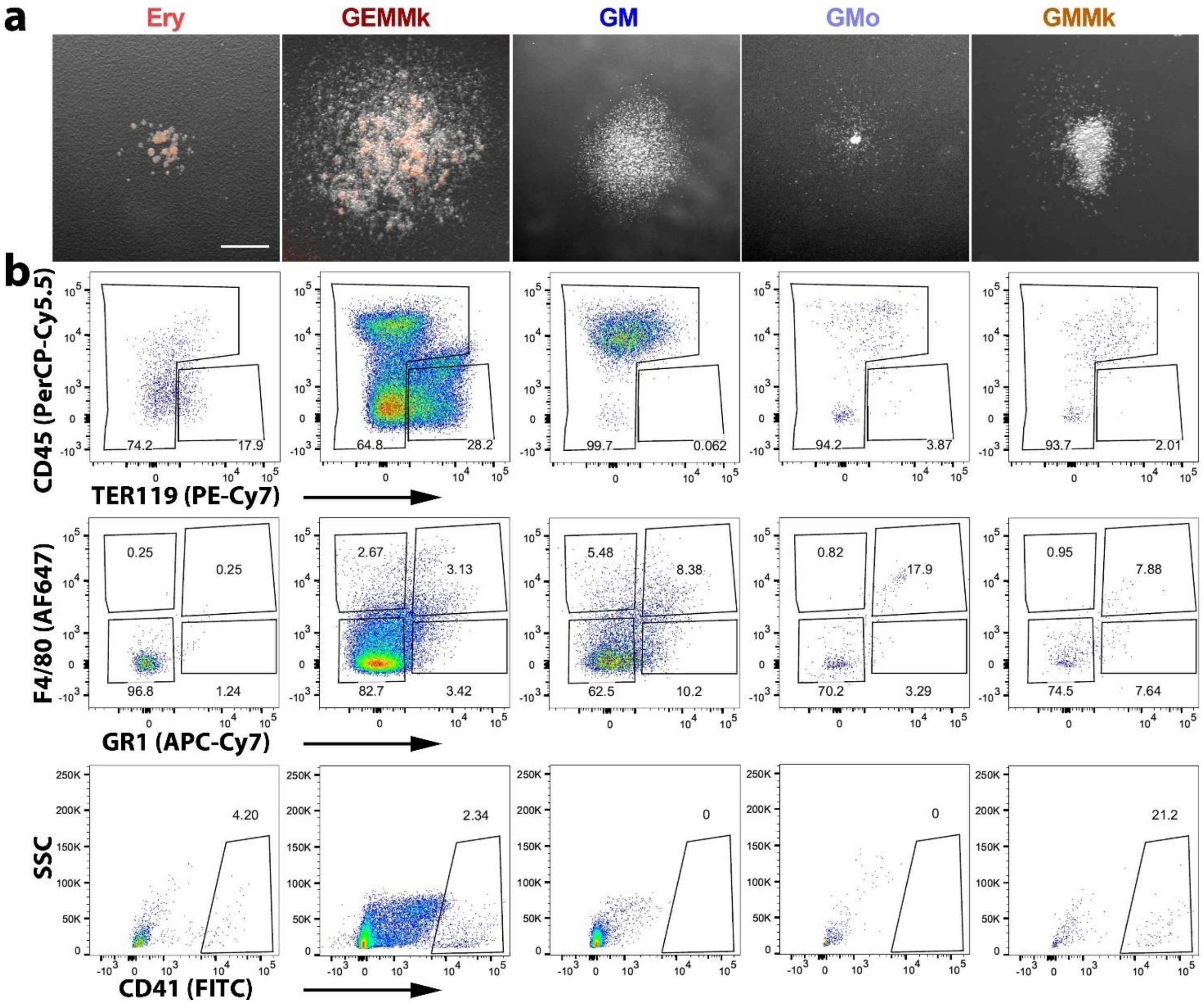

Figure S1. CFU-C assay.

Representative images (**a**) and flow cytometry analysis (**b**) of colonies in the CFU-C assay. Ery: erythroid; GEMMk: granulocyte, erythroid, monocyte/macrophage, megakaryocyte; GM: granulocyte, monocyte/macrophage; GMo: granulocyte, monocyte; GMMk: granulocyte, monocyte/macrophage, megakaryocyte.

Supplementary figure 2

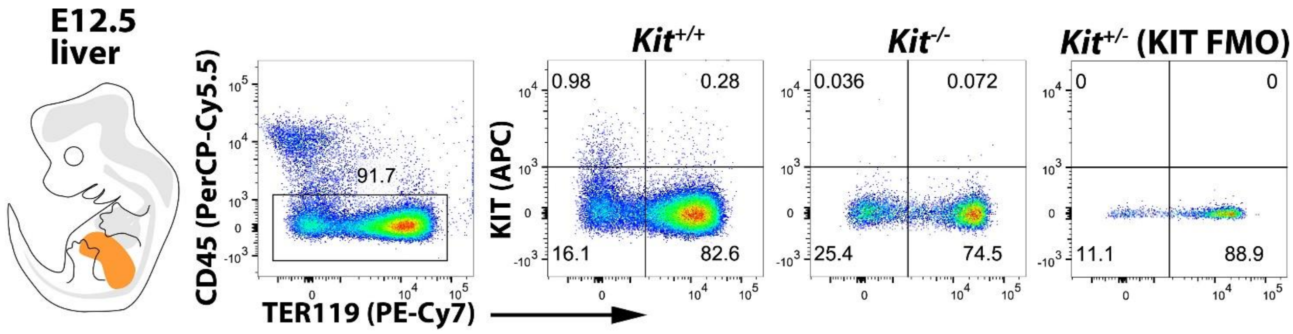

Figure S2. KIT expression in CD45-negative cells in the E12.5 liver.

Flow cytometry analysis of single living cells from E12.5 fetal mouse livers with the indicated markers. The boxes in the left-hand scatter plot indicate the gates used to generate the adjacent scatter plots from the indicated genotypes (n = 5 livers); FMO, fluorescence minus one.

Supplementary figure 3

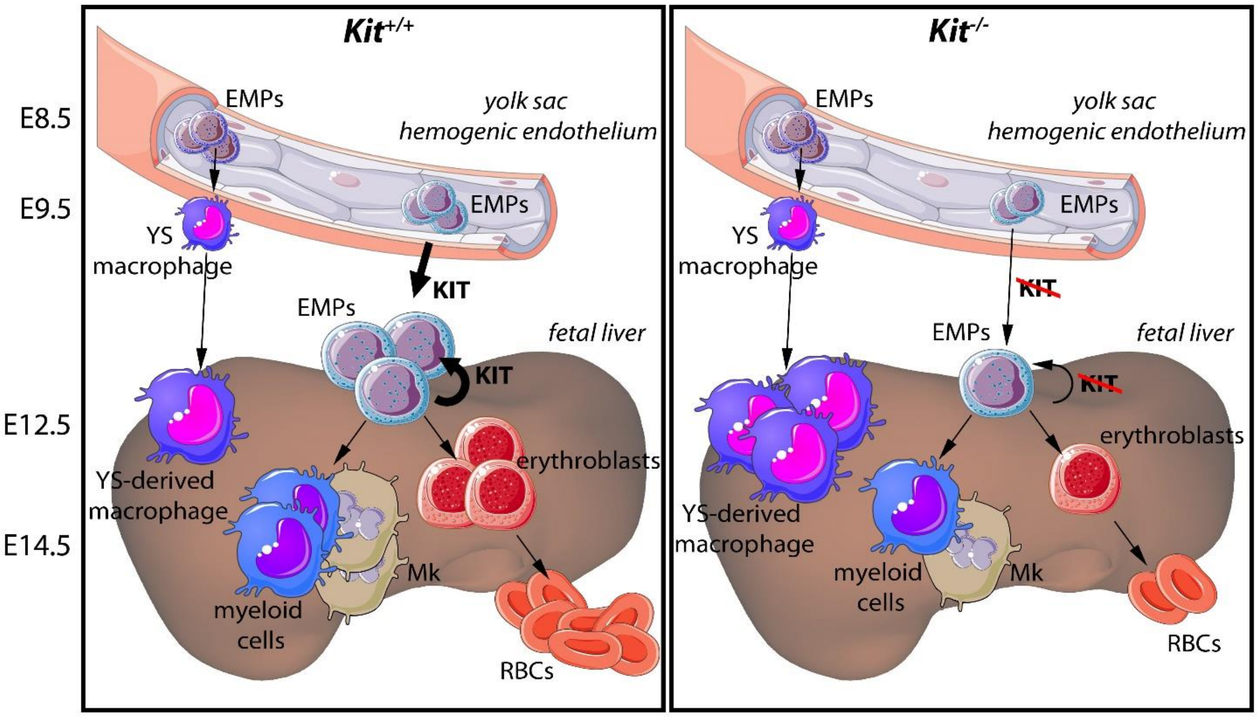

Figure S3. Graphic summary of the working model for KIT function in fetal hematopoiesis.

In the wild type mouse (left panel), EMPs arise in the yolk sac between E8.5 and E9.5. They initially contribute tissue macrophages to the fetal liver and then colonize the fetal liver to establish transient definitive erythropoiesis by E12.5. Starting on E14.5, fetal liver EMPs generate monocytes that will outnumber the initial macrophage population. KIT promotes the expansion of EMPs both in the yolk sac and fetal liver.

In KIT-deficient mice (right panel), the number of liver EMPs is reduced at E12.5, thus impairing erythroblast formation for the generation of red blood cells (RBCs). The number of other progeny from fetal liver-resident EMPs, including megakaryocytes and monocyte-derived macrophages, is also likely reduced. However, the reduced pool of EMPs and EMP-derived erythroblasts did not decrease liver cellularity due to the expansion of yolk sac-derived liver macrophages.
